# Intraoperative Navigation einer Distraktionsverletzung der BWS bei schwersten skoliotischen Veränderungen

**DOI:** 10.1007/s00113-024-01434-0

**Published:** 2024-04-26

**Authors:** Simon Schramm, Johannes Groh, Johannes Krause, Mario Perl

**Affiliations:** Unfallchirurgische und Orthopädische Klinik, Universitätsklinikum Erlangen, Krankenhausstr. 12, 91054 Erlangen, Deutschland

**Keywords:** Navigation, Distraktionsverletzung, Polytrauma, 3D-Bildgebung, Wirbelsäulentrauma, Navigation, Distraction injury, Multiple trauma, 3D imaging, Spinal trauma

## Abstract

Geschildert wird der Fall eines 43-jährigen Patienten, welcher sich bei einem Verkehrsunfall mehrere Verletzungen, u. a. eine Distraktionsverletzung an der Brustwirbelsäule, zuzog. Besonderheit war hier die bestehende Spondylodese mit Materialbruch und sekundärem Repositionsverlust. Aufgrund dessen wurden bei fehlender Einstellbarkeit der Pedikel und abnormem Schraubenkorridor die Führungsdrähte der Pedikelschrauben navigiert gesetzt. Hierdurch kann eine optimale Positionierung mit damit verbundener Patientensicherheit garantiert werden.

## Abstract (en)

The case of a 43-year-old male patient is described, who suffered several injuries due to a traffic accident, including a distraction injury to the thoracic spine. A specific feature of this case was the existing spondylodesis with material fracture and secondary loss of reduction. Due to this, the guidewires of the pedicle screws were placed in a navigation pattern in the absence of adjustable pedicles and an abnormal screw corridor. This guarantees an optimal positioning with associated patient safety.

## Einleitung

Verletzungen der Wirbelsäule haben bei der Schwerstverletztenversorgung einen hohen traumatologischen Stellenwert. So weisen laut Jahresbericht 2022 – TraumaRegister DGU® 29,6 % (*n* = 26.199) der PatientInnen aus dem Basiskollektiv eine Wirbelsäulenverletzung auf [1]. Insbesondere im Bereich der Brustwirbelsäule hat sich in den letzten Jahren die Thematik der Navigation und Robotik etabliert. Eine PubMed-Suche mit den beiden Begriffen „navigation spine“ gibt für die Jahre 2011 und 2012 161 Ergebnisse, während die gleiche Suche im Zeitraum 2021 und 2022 573 passende Treffer hervorbringt. Die Studienlage beruht hier jedoch zumeist auf Normvarianten der Wirbelsäule. Doch bei Verletzungen der voroperierten, skoliotischen Wirbelsäule steht das Traumateam vor besonderen Herausforderungen. Eine solche Kasuistik wird im Folgenden beschrieben.

## Anamnese

Ein 43-jähriger Patient wurde luftgebunden in den Schockraum unseres ÜTZ gebracht. Vorausgegangen war ein Frontalzusammenstoß zweier Pkw, wobei der Beifahrer des Verletzten am Unfallort verstarb. Nach langer technischer Bergung und bei progredienter Somnolenz erfolgte die Schutzintubation des Patienten vor Ort. Fremdanamnestisch nach Auskunft des Notarztes sowie des Rettungsdienstes habe der Patient alle Extremitäten spontan bewegt und zeigte zu keiner Zeit ein peripher neurologisches Defizit. Nach erfolgtem Schockraummanagement nach ATLS® mit Primary und Secondary Survey sowie Durchführung eines Spiral-CT wurden folgende Diagnosen erhoben:

1. Distraktionsverletzung BWK 7/8 mit Materialversagen nach dorsaler Instrumentierung bei schwerer Skoliose (AO Spine B1),
2. mehrfragmentäre Claviculafraktur links (AO 15.2C),
3. Impressionsfraktur am Corpus sterni (AO 16.3.2B),
4. Rippenserienfraktur rechts (Rippen III, V, VI, VII),
5. Fraktur 2. Rippe links mit kleinem Pneumothorax,
6. dislozierte mehrfragmentäre Femurschaftfraktur links (AO 32C3j),
7. Tibiakopftrümmerfraktur links (AO 41C3.3),
8. Pilon-tibiale-Fraktur rechts (AO 43C3.2),
9. Fraktur der Fibula rechts (Weber C),
10. Fraktur des Talus rechts (AO 81.1.B),
11. Abdominalverletzung mit blutigem Aszites bei Peritonealdialyse mit Dünndarmperforation, Hernia umbilicalis ohne Einklemmung und ohne Gangrän,
12. eingeblutete Bursa olecrani links.

Es bestand die Indikation zur Notfalllaparoskopie und zur Stabilisierung der Extremitätenfrakturen mittels Fixateur externe im Sinne der „damage control“. Postoperativ wurde der Patient auf der interdisziplinäre operative Intensivstation betreut.

Zudem erfolgte nun die Einholung der Vorbefunde des Patienten. Laut der externen Dokumentation wurde 2005 ex domo eine dorsale Distraktionsspondylodese nach Zielke bei einer idiopathischen progredienten Segmentskoliose der Segmente Th3–L3 durchgeführt. Bei einem sekundären Korrekturverlust wurde ein Stab bereits im Jahr 2009 gekürzt und partiell Material entfernt. Biomechanisch kam es nun aufgrund des Traumamechanismus mit sternovertebraler Kombinationsverletzung zu einem Materialbruch der dorsalen Stabilisierung.

**Figure 1:**
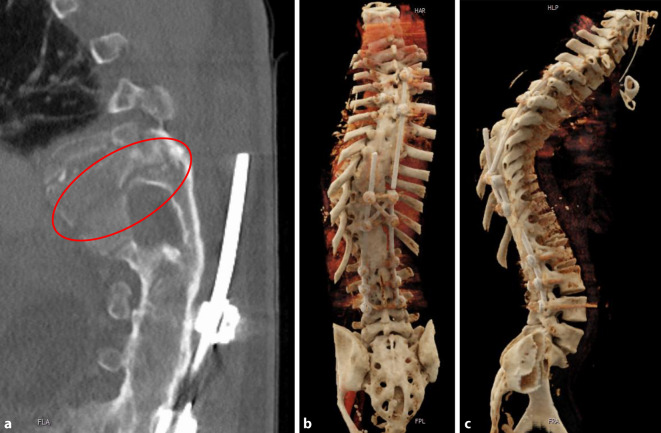

**Figure 2:**
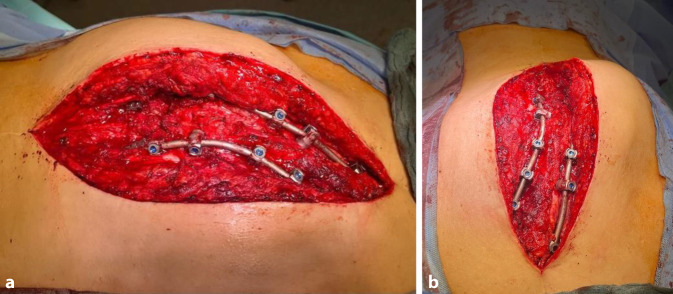

Nach Stabilisierung des Patienten wurde im Rahmen der sekundären Versorgungsphase die Stabilisierung der Distraktionsverletzung an der BWS am 15.12.2022 durchgeführt. Die ausgeprägte skoliotische Fehlstellung nach Korrekturverlust (Skoliosewinkel nach Cobb von 70°) und die markante knöcherne Spondylodese (maximale Dicke der knöchernen Spange: 5 cm) stellten hier die großen Herausforderungen dar. Dies wird in Abb. 1 insbesondere an den „Cinematic-rendering“-Aufnahmen [2] deutlich. Aufgrund der Gesamtsituation mit gebrochenem Material und komplexen Trajektorien wurde sich gegen ein minimal-invasives Verfahren entschieden. Es konnte zunächst bis auf 2, nicht weiter störende, vollständig überbaute Laminahaken das Osteosynthesematerial an der oberen BWS vollständig entfernt, der noch intakte Stab gekürzt und der Fixateur interne an der LWS belassen werden. Mittels Fluoroskopie waren die Pedikel der oberen BWS aufgrund der ausgeprägten knöchernen Spange nicht darstellbar. Hier wurde eine intraoperative Navigation (Fa. Brainlab Curve, Brainlab AG, Olof-Palme-Straße 9, 81829 München, Deutschland) anhand eines intraoperativen 3D-Scans (Fa. Siemens, Cios Spin, Siemens Healthineers AG, 91301 Forchheim, Deutschland) verwendet. Es konnten alle Pedikelschrauben (Fa. Medtronic, Solera) trotz der atypischen Schraubenposition aufgrund der thorakalen Skoliose (Abb. 2) zügig und präzise analog der präoperativen Planung über insgesamt 5 Höhen gesetzt werden, was durch intraoperativen 3D-Scan verifiziert werden konnte. Nachfolgend erfolgte mittels Seit-zu-Seit-Verbinder der Anschluss an das vorhandene Schrauben-Stab-System, um eine Hypermobilität im thorakolumbalen Übergang zu verhindern und keine Veränderung der Hebelkräfte im Vergleich zum präoperativen Befund zu bewirken.

**Figure 3:**
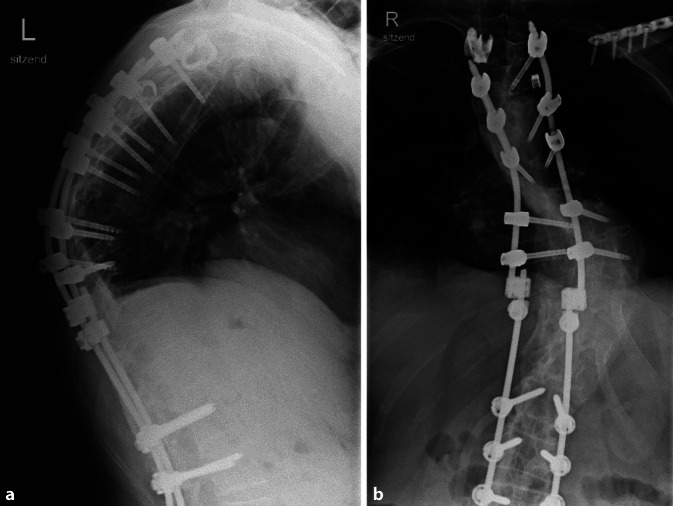

Nach Marknagelosteosynthese des linken Femurs und Doppelplattenosteosynthese des linken Tibiakopfes am 27.12.2022 erfolgte die Verlegung auf die unfallchirurgische Normalstation am 28.12.2022. Die operative Stabilisierung des rechten Sprunggelenks und der linken Clavicula wurde am 05.01.2023 durchgeführt. Nach insgesamt 44 Tagen erfolgte am 24.01.2023 die Verlegung in die Frührehabilitation weiterhin ohne peripher neurologisches Defizit sowie Blasen- oder Mastdarmstörungen. Auch die nachfolgenden Verlaufskontrollen zeigen insbesondere im Bereich der Brustwirbelsäule einen regelrechten Verlauf (Abb. 3).

## Diskussion

Neben der Herausforderung der komplexen Revisionsstabilisierung und der damit verbundenen Wahl des geeigneten Stabilisierungsverfahrens und -umfanges wird anhand dieses Falles zudem deutlich, dass die intraoperative Navigation die Platzierung der Pedikelschrauben auch für ein sehr erfahrenes Wirbelsäulenteam erleichtert. Viele Studien beschreiben die Vorteile eines Hybrid-OP mit der genaueren und zügigeren Schraubenplatzierung [3], oder vergleichen Navigation mit der Freihandplatzierung unter Bildwandlerkontrolle [4]. Letztendlich wird die Sicherheit der PatientInnen durch eine bessere Schraubenlage verbessert [5].

Bei einer ausgeprägten Skoliose sowie komplexen Voroperationen bedarf es nochmals mehr Erfahrung durch den Operateur, da in diesen Fällen die Trajektorien der Pedikelschrauben atypisch verlaufen können und teilweise deutlich vom Gewohnten abweichen [6, 7]. Hier kommt der Navigation und auch in Zukunft der Robotik eine tragende wichtige Rolle zu [8]. Aufgrund der Komplexität der fluoroskopischen Einstellung der Pedikel kann hier durch Navigation eine deutliche Strahlenreduktion für Patient und Chirurgen erzielt werden [9].

## Fazit für die Praxis

Bei komplexen anatomischen Besonderheiten oder Voroperationen bringt die intraoperative Navigation oder Robotik einen erheblichen Mehrwert. Bei komplett aufgehobener Möglichkeit zur Darstellung der Pedikel und stark abweichenden Trajektorien bietet sie sowohl den ChirurgInnen als auch den PatientInnen eine dringend benötigte Sicherheit.
